# Association between bone-specific physical activity scores and pQCT-derived measures of bone strength and geometry in healthy young and middle-aged premenopausal women

**DOI:** 10.1007/s11657-018-0495-8

**Published:** 2018-07-28

**Authors:** SoJung Kim, Breanne S. Baker, Pragya Sharma-Ghimire, Debra A. Bemben, Michael G. Bemben

**Affiliations:** 1 Department of Physical Therapy and Kinesiology, University of Massachusetts Lowell, 3 Solomont Way, Lowell, MA 01854 USA; 2 Bone Density Research Laboratory, Department of Health and Exercise Science, University of Oklahoma, 1401 Asp Avenue, Norman, OK 73019 USA; 3 Department of Physical Education and Exercise Studies, Lander University, 320 Stanley Ave, Greenwood, SC 29649 USA

**Keywords:** Bone mineral density, Bone quality, Hip structural analysis, pQCT

## Abstract

**Summary:**

The aim of this study was to determine if bone-specific physical activity questionnaire (BPAQ) scores were positively related to bone health in healthy young and middle-aged premenopausal women. The total BPAQ was a stronger predictor of bone strength and bone mineral density of hip in young women as compared to middle-aged premenopausal women.

**Purpose:**

The purpose of this study was to determine whether the BPAQ scores were predictive indices of volumetric BMD (vBMD), bone strength, and bone geometry in young and middle-aged premenopausal women.

**Methods:**

Healthy young (*n* = 60) and middle-aged premenopausal women (*n* = 54) between the ages of 18 and 50 years were recruited for this study. Areal bone mineral density (aBMD) of lumbar spine and dual proximal femur (FN; femoral neck) was measured using DXA. We assessed vBMD of the tibia 4%, 38%, and 66% by peripheral quantitative computed tomography (pQCT). The BPAQ was used to obtain a comprehensive account of lifetime physical activity related to bone health.

**Results:**

Pearson’s correlation tests showed positive correlations between total BPAQ and aBMD of the right FN (*r* = 0.313, *p* = 0.015) and the left FN (*r* = 0.307, *p* = 0.017) in young women while not found in middle-aged premenopausal women (*p* > 0.05). A positive relationship was only observed between total BPAQ and tibia 38% vBMD in middle-aged premenopausal women (*r* = 0.283, *p* = 0.038). All bone geometry variables were associated with total BPAQ (*r* = 0.280–0.422, *p* = 0.03–0.001) in young women. The Strength-Strain Index of tibia 38% (*r* = 0.350, *p* = 0.006) and 66% (*r* = 0.406, *p* = 0.001) was associated with total BPAQ in young women. In both young and middle-aged premenopausal women, when age, bone-free lean body mass (BFLBM), and total BPAQ were included in a stepwise multiple linear regression analysis, BFLBM was a significant predictor of all aBMD variables, accounting for 7–25.7% (*p* = 0.043–0.001).

**Conclusions:**

The total BPAQ score-derived physical activity was more predictive of positive bone characteristics in young women than in middle-aged premenopausal women.

## Introduction

Bone-strengthening activities (e.g., jumping, running, and gymnastics) have been shown to positively affect bone accrual in growing children [1, 2] and overall bone health in later life [3]. Bone-loading forces from moderate to high intensity in parallel with increasing percent of maximal heart rate or 1 repetition maximum (RM) are recommended to help preserve bone health during adulthood [3]. Numerous studies have utilized various assessment tools (e.g., questionnaires, pedometers, accelerometers) to predict bone strength in both clinical and healthy populations [4–7], but recently the most common methods for assessing one’s historical bone-loading exercises that affect bone strength have been criticized in different bone quality components as well as different age groups.

Among physical activity questionnaires, the bone-specific physical activity questionnaire (BPAQ) is becoming more widely used to estimate bone-specific loading exercise history and its relation to areal bone mineral density (aBMD) as measured by dual-energy X-ray absorptiometry (DXA). The BPAQ algorithms were developed based on lifetime recreational sports and physical activities that involve ground reaction force (GRF)-derived loading values [4]. Previous findings have shown its positive associations with the aBMD of femoral neck, total hip, lumbar spine, and whole body in healthy young adults [4, 8, 9] and middle-aged older men [10]. In contrast, its relationships with bone strength and architecture have been controversial in different trials. Weeks and Beck [4] reported that past and current BPAQ scores had significant ability to detect bone strength parameters whereas other traditional measures of physical activity (e.g., Bone Loading History Questionnaire; BLHQ, 3-day Physical Activity Record; 3DPAR, Pedometer) did not. Significant associations between the BPAQ and magnetic resonance imaging (MRI)-derived midtibia cortical bone quality were also detected in adolescent females [9]. Rantalainen et al. [11] found that higher scores of BPAQ had more bone mass and robust bone geometry compared to those with lower BPAQ scores. However, Farr et al. [6] found that the modified past year physical activity questionnaire (PYPAQ) predicted better indices of bone strength compared to BPAQ, 3DPAR, and pedometer use in girls.

The BPAQ algorithms have been shown to be related to aBMD, but its relations to volumetric BMD (vBMD), bone strength, and bone geometry as measured by peripheral quantitative computed tomography (pQCT) have not been well studied in healthy young and middle-aged premenopausal women. Therefore, the purpose of this cross-sectional study was to determine whether the BPAQ scores were predictive indices of bone characteristics (vBMD, bone strength, and bone geometry) in young and middle-aged premenopausal women. We hypothesized that there would be positive associations between total BPAQ scores and measures of vBMD and bone quality obtained by pQCT. Also, we hypothesized that these significant relationships would be consistently found in young and middle-aged premenopausal women.

## Methods

### Participants

Healthy young (*n* = 60) and middle-aged premenopausal women (*n* = 54) between the ages of 18 and 50 years were recruited from the University of Oklahoma and the surrounding Oklahoma City metro area. Participants were included if they had regular menstrual cycles and were free of bone disease and not taking any medications (e.g., steroid hormones, calcitonin, or corticosteroids) that affect the skeletal systems. Volunteers who were outside of the 18–50 years age range and who exceeded the weight limit of the DXA (300 lbs) were excluded. This study was approved by the University Oklahoma Health Sciences Center Institutional Review Board and written informed consent was obtained from each participant prior to testing.

### Anthropometry and body composition

We measured standing height to the nearest 0.1 cm and weight to the nearest 0.1 kg using a wall stadiometer and an electronic scale (Tanita BWB-800, IL, USA), respectively. We used DXA (Lunar Prodigy, GE Medical Systems, encore 2002 Software, version 10.50.086) with standard positioning for a total body scan to assess body composition of the whole body. We obtained measures of bone-free lean body mass (BFLBM, kg), fat mass (kg), and percent (%) fat from the total body DXA scan. The same three qualified technicians performed daily calibrations and conducted all measures following standard manufacturer’s procedures. After removing all metal, plastic objects, or other high-density objects associated with the participant’s clothes, participants were asked to lie down on the DXA table in the supine position. The participant’s shoulders and hips were centered, and the hands were placed by the side of the legs. Velcro straps were placed around the knees and ankles to hold feet together for the duration of the scan. Scan modes were determined by the software based on the truncal thickness: thick > 25 cm, standard 13–25 cm, and thin < 13 cm. In our laboratory, the coefficient of variation (CV%) for BFLBM, fat mass, and % fat ranged from 1.3 to 1.9%.

### Questionnaires

All participants completed a menstrual history questionnaire, which was used to gather information regarding pregnancy, cycle characteristics, and contraceptive use; women who reported cycle irregularities or menopause symptoms were excluded. A health history questionnaire was utilized to examine any medical history that could affect bone health. Daily calcium intake (mg/day), including supplements, was assessed by a food frequency questionnaire [12].

For the BPAQ, the participants were asked to fill out two independent sections. The past period (pBPAQ) constituted any activity reported from 1 year of age to 12 months previous to testing. The current period (cBPAQ) included any activity reported from the past 12 months and the total period (tBPAQ) was calculated as the average of pBPAQ and cBPAQ scores. In this present study, we only used tBPAQ scores. Qualified researchers administrated and analyzed all values using an online BPAQ calculator (www.fithdysign.com/BPAQ/). It has been previously reported that intra-class correlation coefficients for inter- and intra-tester reliability for the BPAQ measures are very high (0.92 and 0.97, respectively) [13]. Algorithms used to analyze BPAQ responses have been described in detail [4].

### Areal bone mineral density and hip structural analysis

We used DXA (Lunar Prodigy, GE Medical Systems, encore 2002 Software, version 10.50.086) to assess aBMD of lumbar spine (L1-L4) and dual proximal femur (total hip, femoral neck). For the lumbar spine measurement, a block-shaped cushion was placed under the participant’s feet in order to obtain the highest quality and most accurate lumbar spine images. The qualified technicians adjusted the positioning laser crosshairs to approximately 5 cm below the umbilicus to include part of L5, some of the iliac crest, and part of T12 with some rib. For the dual proximal femur, the participant’s feet were secured to the DualFemur™ positioner to maintain the appropriate internal rotation of the femur. The positioning laser was moved to a position 4 cm inferior to the greater trochanter or 1 cm inferior to the pubic symphysis in the midline of the thigh. The dual proximal femur scan included large and rounded, with soft tissue seen above the superior edge of the bone. Additionally, hip structural analysis (HSA) using the proximal femur scans to measure both the aBMD of the hip and the structural geometry of the cross-sections traversing the proximal femur allowing for the determination of the hip strength index, buckling ratio, cross-sectional moment of inertia (CSMI), and section modulus [14] was completed. The CV% for the aBMD of lumbar spine (L1-L4), dual total hip, and dual femoral neck ranged from 0.68 to 1.39%.

### Volumetric bone mineral density, bone geometry, and bone strength

We used pQCT (XCT 3000, Software version 6.00, Stratec Medizintechnik, GmbH, Pforzheim, Germany) to acquire tibia vBMD and geometry variables. The same three qualified pQCT technicians completed all the quality assurance scans each day. For the tibia scans (4%, 38%, and 66%), the participants were asked to cross the non-dominant leg over the other leg and pQCT technicians measured the length of the tibia from the medial malleolus to the tibial plateau. The participants were asked to remain still and to breathe normally during the scanning process. After the scout view was displayed, a reference line was set at the exact location of the distal tibial plateau. We excluded tibia scans with the motion artifacts using a grading scale and attempted a rescan if needed. We used a voxel size of 0.4 mm for all sites at the scout view speed of 40 mm/s and CT speed of 20 mm/s, respectively. We used contour mode 3 at 169 mg/cm^3^ and peel mode 4 at 650 mg/cm^3^ with a 10% peel for the distal tibia 4% to determine total vBMD, total bone area, and trabecular area. We used cort mode 2 at 710 mg/cm^3^ to define cortical results at the distal tibia 38% and 66%. The Strength-Strain Index (SSI) was obtained using the cort mode 2 at 480 mg/cm^3^. In our Bone Density Research Lab, the in vivo precision CV% for the pQCT bone measurements ranged from 0.57 to 0.83% at the 4% tibia; from 0.31 to 1.21% at the 38%; and from 0.50 to 0.95% at the 66% tibia.

### Data analyses

We performed all analyses using SPSS for Mac version 24 (SPSS Inc., Chicago, IL, USA) and data are reported at mean ± SD. We used scatter plots and box plots to identify possible data errors and outliers prior to data analyses. We compared descriptive characteristics and bone variables between young and premenopausal participants using Student’s *t* tests. Pearson’s correlation tests were used to identify relationships between measures of aBMD and vBMD and total BPAQ scores in young and middle-aged premenopausal women separately. We included BFLBM, age, and total BPAQ scores as covariates in stepwise multiple regression models to determine the variables that predict variance in aBMD and vBMD, respectively. We tested collinearity statistics to check if BFLBM, age, and total BPAQ were strongly related. We found that they were not related to one another. We set the level of significance at *p* < 0.05.

## Results

Table 1 shows physical characteristics, body composition, daily calcium intake, and total BPAQ score for each group. There were no significant differences in weight, BMI, and total BPAQ scores between young and middle-aged premenopausal women (*p* > 0.05). We found a significant age difference between groups and young women were taller (5.6 cm), had greater BFLBM (3.0 kg), and less fat mass (− 4.0 kg) and lower % fat (− 3.6%) than middle-aged premenopausal women (*p* < 0.05).

**Table 1:** Descriptive data for the study population (means ± SD)

	Young women (*n* = 60)		Middle-aged premenopausal women (*n* = 54)		
	Mean ± SD	Range	Mean ± SD	Range	Significance
Age (years)	22.6 ± 3.1	18.0–30.2	42.3 ± 4.5	35.6–50.9	0.001
Height (cm)	168.3 ± 5.6	154.1–180.0	162.8 ± 5.4	150.0–176.0	0.001
Weight (kg)	68.9 ± 11.7	49.6–112.9	68.0 ± 11.5	46.7–99.3	0.656
BMI (kg/m^2^)	24.3 ± 3.6	18.3–36.7	25.7 ± 4.5	18.2–39.5	0.073
BFLBM (kg)	42.6 ± 6.7	30.1–57.8	39.6 ± 5.0	29.5–52.5	0.008
Fat mass (kg)	22.9 ± 7.8	12.0–51.5	26.6 ± 10.6	10.2–67.4	0.039
% fat	33.5 ± 6.4	22.2–47.1	37.1 ± 8.8	15.1–53.5	0.015
Calcium (mg/day)	797.3 ± 367.3	190.0–1960.0	1014.3 ± 566.7	247.0–3244.0	0.016
Total BPAQ	33.6 ± 25.3	0.3–98.2	27.1 ± 24.9	1.1–84.7	0.172

*SD* standard deviation, *BMI* Body Mass Index (kg/m^2^), *BFLBM* bone-free lean body mass (kg), *% fat* total body fat percentage

Young women had significantly greater values for 15 of the 21 aBMD and vBMD variables measured including the L1-L4 and both left and right total hip and femoral neck (*p* < 0.05) as compared to middle-aged premenopausal women. Young women also had greater values for all tibia sites measures (*p* < 0.05). At the 38% site, only cortical area and SSI were greater in young women as compared to middle-aged premenopausal women; while at the 66% site, young women had greater total vBMD, cortical thickness, cortical area, and SSI (*p* < 0.05). There were no significant differences in tibia 38% (total vBMD, cortical vBMD, cortical thickness, total bone area) and tibia 66% (cortical vBMD, total bone area) between groups (*p* > 0.05). The results of structural parameters of HSA showed that young women had higher values in dominant and non-dominant legs’ hip strength index, section modulus, and CSMI (*p* < 0.05) as compared to middle-aged premenopausal women. There was a significant difference in buckling ratio, showing that young women had lower values in both dominant and non-dominant leg (*p* = 0.001). The post hoc power results for primary outcomes (vBMD, bone strength, and geometry) ranged from 0.47 to 0.99 (Table 2).

**Table 2:** Study population aBMD and vBMD measures (means ± SD)

	Young women (*n* = 60)	Middle-aged premenopausal women (*n* = 54)	
	Mean ± SD	Mean ± SD	Significance
aBMD (g/cm^2^)			
Right total hip	1.085 ± 0.118	1.018 ± 0.112	0.002
Right femoral neck	1.098 ± 0.125	0.994 ± 0.112	0.001
Left total hip	1.081 ± 0.116	1.014 ± 0.112	0.002
Left femoral neck	1.091 ± 0.121	0.995 ± 0.115	0.001
L1-L4	1.273 ± 0.133	1.224 ± 0.127	0.048
4% tibia			
Total vBMD (mg/cm^3^)	314.297 ± 36.608	292.222 ± 34.955	0.001
Trabecular vBMD (mg/cm^3^)	265.272 ± 33.279	234.109 ± 27.140	0.001
Total bone area (mm^2^)	1000.47 ± 114.71	926.19 ± 116.89	0.001
Trabecular area (mm^2^)	834.07 ± 106.21	785.35 ± 113.06	0.019
38% tibia			
Total vBMD (mg/cm^3^)	948.067 ± 52.415	937.724 ± 64.374	0.347
Cortical vBMD (mg/cm^3^)	1192.863 ± 16.523	1193.407 ± 26.701	0.898
Cortical thickness (mm)	5.55 ± 0.59	5.34 ± 0.60	0.057
Total bone area (mm^2^)	364.51 ± 40.46	353.29 ± 39.10	0.136
Cortical area (mm^2^)	278.12 ± 33.67	264.08 ± 29.88	0.021
Strength-Strain Index (mm^3^)	1554.6 ± 247.3	1451.6 ± 200.2	0.016
66% tibia			
Total vBMD (mg/cm^3^)	712.192 ± 58.856	674.746 ± 69.706	0.002
Cortical vBMD (mg/cm^3^)	1154.382 ± 18.025	1145.602 ± 29.655	0.063
Cortical thickness (mm)	4.38 ± 0.49	4.05 ± 0.54	0.001
Total bone area (mm^2^)	527.95 ± 64.42	517.78 ± 60.84	0.389
Cortical area (mm^2^)	295.35 ± 36.04	272.58 ± 31.95	0.001
Strength-Strain Index (mm^3^)	2341.3 ± 385.4	2160.2 ± 324.7	0.008
Dominant leg			
Hip strength index	1.62 ± 0.36	1.47 ± 0.35	0.029
Buckling ratio	2.45 ± 0.77	3.28 ± 1.25	0.001
Section modulus (mm^3^)	668.07 ± 128.90	600.36 ± 95.32	0.002
CSMI (mm^4^)	9981.58 ± 2587.85	9098.92 ± 1858.13	0.043
Non-dominant leg			
Hip strength index	1.64 ± 0.32	1.46 ± 0.33	0.004
Buckling ratio	2.57 ± 0.92	3.23 ± 1.03	0.001
Section modulus (mm^3^)	665.18 ± 116.91	593.78 ± 97.28	0.001
CSMI (mm^4^)	9934.62 ± 2230.93	9135.23 ± 1807.07	0.041

Dominant and non-dominant leg indicates premenopausal women (*n* = 52)

*SD* standard deviation, *L1-L4* lumbar spine 1–4, *CSMI* cross-sectional moment of inertia

**Fig. 1:**
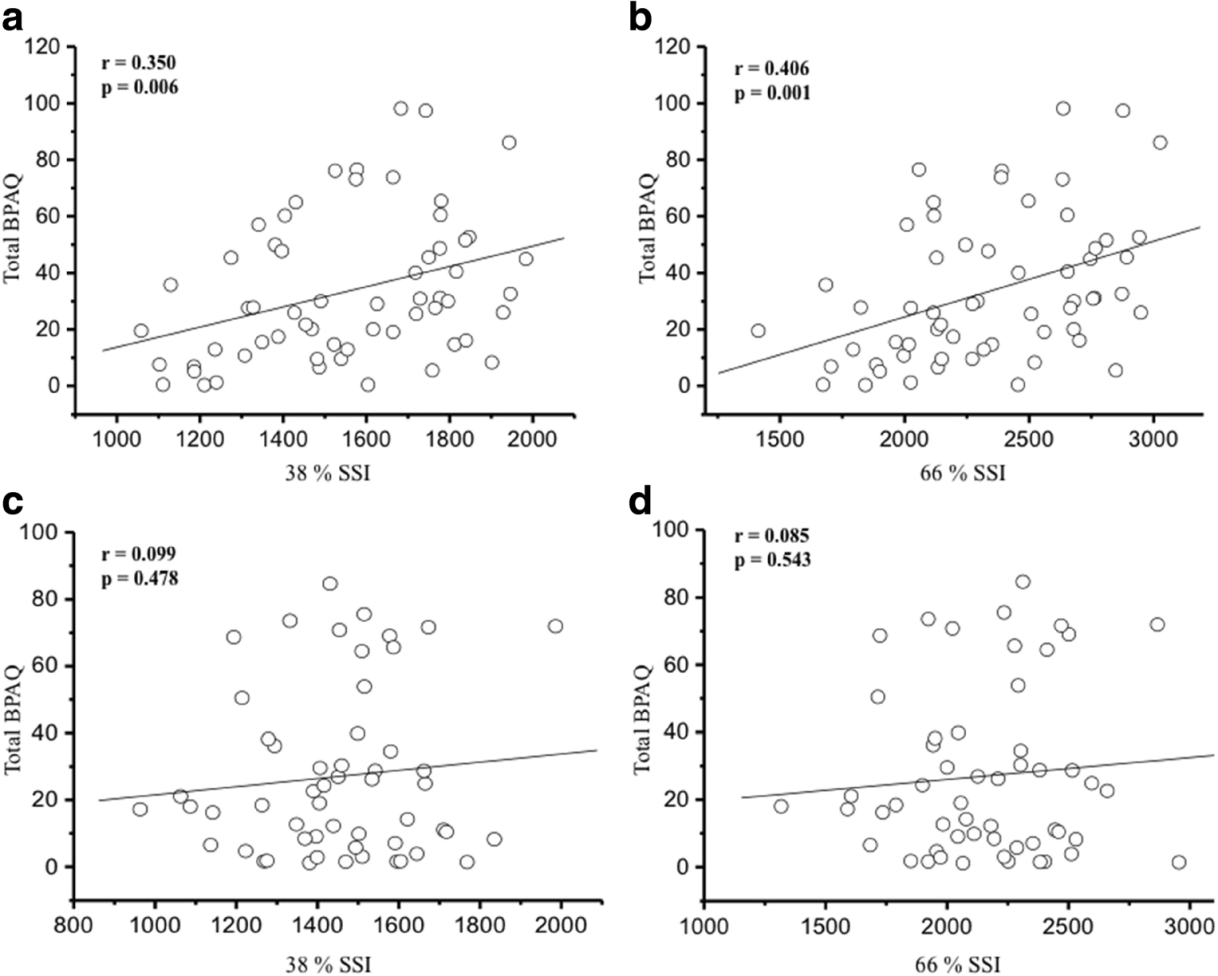
Correlation of total BPAQ scores with 38% and 66% Strength-Strain Index (*N* = 114). SSI, Strength-Strain Index; BPAQ: tBPAQ, total bone-specific physical activity questionnaire. **a** tBPAQ score with 38% SSI. **b** tBPAQ score with 66% SSI in young women. **c** tBPAQ score with 38% SSI. **d** tBPAQ score with 66% SSI in middle-aged premenopausal women

There were positive correlations between total BPAQ scores and aBMD of right femoral neck (*r* = 0.313, *p* = 0.015) and left femoral neck (*r* = 0.307, *p* = 0.017) in young women while no significant relationships were found in middle-aged premenopausal women (*p* > 0.05). A positive relationship was only observed between tBPAQ score and tibia 38% vBMD in middle-aged premenopausal women (*r* = 0.283, *p* = 0.038). None of the vBMD variables were correlated with total BPAQ scores in young women. All bone size variables were associated with total BPAQ score (*r* = 0.280–0.422, *p* = 0.03–0.001) in young women while there was only one positive relationship between tibia 38% cortical thickness and total BPAQ score in middle-aged premenopausal women (*r* = 0.273, *p* = 0.046) (Table 3). There were no significant associations between total BPAQ scores and structural parameters of HSA in middle-aged premenopausal women (*p* > 0.05). However, young women had negative associations between total BPAQ and CSMI in both legs and buckling ratio and section modulus in non-dominant leg (*p* < 0.05) (Table 4). The SSI of tibia 38% (*r* = 0.350, *p* = 0.006) and 66% (*r* = 0.406, *p* = 0.001) was associated with total BPAQ scores in young women while no relationships were found in middle-aged premenopausal women (Fig. 1).

**Table 3:** Pearson’s correlation coefficients between total BPAQ and aBMD and vBMD measures

	Young women (*n* = 60)		Middle-aged premenopausal women (*n* = 54)	
	*r*	Significance	*r*	Significance
aBMD (g/cm^2^)				
Right total hip	0.190	0.145	0.186	0.179
Right femoral neck	0.313	0.015	0.260	0.057
Left total hip	0.175	0.180	0.211	0.125
Left femoral neck	0.307	0.017	0.227	0.098
L1-L4	0.094	0.476	0.236	0.086
4% tibia				
Total vBMD (mg/cm^3^)	− 0.018	0.891	0.199	0.149
Trabecular vBMD (mg/cm^3^)	0.009	0.943	0.220	0.110
Total bone area (mm^2^)	0.365	0.004**	− 0.003	0.983
Trabecular area (mm^2^)	0.311	0.016*	− 0.015	0.914
38% tibia				
Total vBMD (mg/cm^3^)	0.131	0.318	0.283	0.038*
Cortical vBMD (mg/cm^3^)	− 0.078	0.555	0.145	0.295
Cortical thickness (mm)	0.280	0.030*	0.273	0.046*
Total bone area (mm^2^)	0.333	0.009**	0.096	0.491
Cortical area (mm^2^)	0.369	0.004**	0.222	0.107
66% tibia				
Total vBMD (mg/cm^3^)	0.081	0.536	0.151	0.275
Cortical vBMD (mg/cm^3^)	− 0.154	0.240	0.004	0.976
Cortical thickness (mm)	0.296	0.022*	0.134	0.333
Total bone area (mm^2^)	0.355	0.005**	0.046	0.743
Cortical area (mm^2^)	0.422	0.001**	0.173	0.210

*aBMD* areal bone mineral density (g/cm^2^), *L1-L4* lumbar spine 1–4, *vBMD* volumetric bone mineral density (mg/cm^3^), *BPAQ* bone-specific physical activity questionnaire

*p < 0.05; **p < 0.01

**Table 4:** Pearson’s correlation coefficients between total BPAQ and hip structural analysis parameters

	Young women (*n* = 60)		Middle-aged premenopausal women (*n* = 52)	
	*r*	Significance	*r*	Significance
Dominant leg				
Hip strength index	− 0.045	0.732	− 0.022	0.877
Buckling ratio	− 0.119	0.365	− 0.159	0.266
Section modulus (mm^3^)	− 0.229	0.078	0.245	0.080
CSMI (mm^4^)	− 0.263	0.043*	0.062	0.662
Non-dominant leg				
Hip strength index	− 0.104	0.428	0.026	0.855
Buckling ratio	− 0.255	0.049*	0.079	0.581
Section modulus (mm^3^)	− 0.266	0.040*	0.225	0.109
CSMI (mm^4^)	− 0.329	0.010*	0.052	0.714

*BPAQ* bone-specific physical activity questionnaire, *CSMI* cross-sectional moment of inertia

*p < 0.05; **p < 0.01

In both young and premenopausal women, when age, BFLBM, and total BPAQ score were included in a stepwise multiple linear regression analysis, BFLBM was a significant predictor of all aBMD variables, accounting for 7–25.7% (*p* = 0.043–0.001). In middle-aged premenopausal women, only total BPAQ score predicted 8% of the variance in tibia 38% total BMD (*p* = 0.038) and 38.7% of the variance in tibia 38% cortical thickness (*p* = 0.001). The rest of vBMD and geometry variables was explained by the BFLBM (14.5–37.8%, *p* = 0.004–0.001). In young women, only BFLMB predicted 7.2–53.0% of the variance in vBMD and geometry variables (*p* = 0.038–0.001) (Table 5).

**Table 5:** Stepwise multiple regression models for aBMD and vBMD Measures

	Young women (*n* = 60)				Middle-aged premenopausal women (*n* = 54)			
	Best significant predictor variable	*β*	SEE	*R* ^2^	Best significant predictor variable	*β*	SEE	*R* ^2^
aBMD (g/cm^2^)								
Right total hip	BFLBM	0.498	0.103	0.248	BFLBM	0.505	0.097	0.255
Right femoral neck	BFLBM	0.507	0.109	0.257	BFLBM	0.369	0.106	0.136
Left total hip	BFLBM	0.497	0.101	0.247	BFLBM	0.482	0.099	0.232
Left femoral neck	BFLBM	0.483	0.107	0.234	BFLBM	0.426	0.105	0.181
L1-L4	BFLBM	0.345	0.126	0.119	BFLBM	0.276	0.123	0.076
4% tibia								
Total vBMD (mg/cm^3^)	NS	NS	NS	NS	NS	NS	NS	NS
Trabecular vBMD (mg/cm^3^)	NS	NS	NS	NS	NS	NS	NS	NS
Total bone area (mm^2^)	BFLBM	0.485	101.194	0.235	BFLBM	0.549	98.615	0.302
Trabecular area (mm^2^)	BFLBM	0.442	96.080	0.195	BFLBM	0.483	99.958	0.233
38% tibia								
Total vBMD (mg/cm^3^)	NS	NS	NS	NS	Total BPAQ	0.283	62.341	0.080
Cortical vBMD (mg/cm^3^)	Age	0.349	15.619	0.122	NS	NS	NS	NS
Cortical thickness (mm)	BFLBM	0.558	0.491	0.311	BFLBM	0.566	0.503	0.320
Total bone area (mm^2^)	BFLBM	0.620	32.028	0.384	BFLBM	0.381	36.492	0.145
Cortical area (mm^2^)	BFLBM	0.704	24.107	0.496	BFLBM	0.615	23.786	0.378
66% tibia								
Total vBMD (mg/cm^3^)	NS	NS	NS	NS	NS	NS	NS	NS
Cortical vBMD (mg/cm^3^)	BFLBM	− 0.269	17.510	0.072	NS	NS	NS	NS
Cortical thickness (mm)	BFLBM	0.501	0.426	0.251	BFLBM	0.427	0.489	0.182
Total bone area (mm^2^)	BFLBM	0.611	51.418	0.374	BFLBM	0.423	55.651	0.179
Cortical area (mm^2^)	BFLBM	0.728	24.921	0.530	BFLBM	0.581	26.252	0.338

*BFLBM* bone-free lean body mass, *SEE* standard error of estimate, *aBMD* areal bone mineral density (g/cm^2^), *L1-L4* lumbar spine 1–4, *vBMD* volumetric bone mineral density (mg/cm^3^)

## Discussion

The aim of this cross-sectional study was to determine if total BPAQ scores were positively related to pQCT-derived measures of bone strength and geometry in healthy young and middle-aged premenopausal women and if these relationships were consistently found in two different age groups. The total BPAQ scores were not significant predictors of vBMD both in young and middle-aged premenopausal women; however, significant relationships were found in bone geometry and bone strength in young women. Also, positive associations between total BPAQ scores and measures of aBMD of dual femoral neck were only found in young women.

BPAQ scores have been shown to have positive associations with aBMD of femoral neck as measured by DXA in adolescent and college-aged females [8, 9] and in young and older men [4, 10]. In the current study, we found consistent positive correlations between total BPAQ scores and aBMD of dual femoral neck in young women (18.0–30.2 years); however, these results were not significant in middle-aged premenopausal women (35.6–50.9 years). Previous studies have indicated that women in their 20s had more positive associations between physical activity and aBMD at the hip and spine [15] as compared to women in their 30s or 40s [16, 17]. It is possible that bone-specific loading exercise would be a significant factor to increase bone mass before the peak bone mass formation which normally occurs around the third decade of life. Ho et al. [18] found that leisure time physical activity was not related to bone mass in women aged 31–40, whereas lean body mass and fat mass were highly related to aBMD. Our study results also demonstrated that BFLBM was a significant predictor of all aBMD sites, accounting for 7–25.7% in middle-aged premenopausal women as well as young women. Other relevant factors such as age, diet, body composition, and health status may be more sensitive to predict bone mass than bone-loading physical activity history estimated by BPAQ scores in middle-aged premenopausal women. We did not detect any relationships between total BPAQ and aBMD of lumbar spine in both age groups. Our findings also supported the previous research that suggests BPAQ scores are not positively related to aBMD of lumbar spine in young women [4, 8]. It seems that the BPAQ algorithm may be more related to aBMD of femoral sites. Our study also analyzed cross-sectional geometrical strength of hip using DXA-based HSA and found negative relationships of total BPAQ scores and HSA parameters (buckling ratio, section modulus, and CSMI) in young women. The magnitude of these associations is relatively weak (*r* = − 0.25 to approximately − 0.32). Weeks et al. [4] did not find any relationships between BPAQ scores and femoral neck CSMI in young men and women; however, current BPAQ scores predicted variance in lumbar spine index of bone structural strength (38%, *p* = 0.005). Our study did not measure bone strength in lumbar spine using DXA.

The BPAQ algorithms were developed from ground reaction forces-derived loading values, which account for lifetime physical activity affecting bone health. A two-dimensional imaging tool, DXA, was used to measure bone outcomes (aBMD) and a small number of participants were only young adults (*N* = 40, mean age = 24.6 years) [4]. It is plausible that BPAQ scores may have limited interpretations across various age groups as well as bone quality parameters (bone strength and bone geometry). Unlike DXA, pQCT uses three-dimensional imaging technology to provide many aspects of bone structure that contribute to bone strength [19]. Bone strength is highly influenced by bone geometry parameters as well as bone-loading physical activity [20, 21]. Much like our aBMD results, total BPAQ scores were significantly related to vBMD measures of tibia bone strength and geometry in young women, but not in middle-aged premenopausal women. Rantalainen et al. [11] also found children and young adults who had higher scores of BPAQ had greater vBMD and robust bone geometry compared to those with lower BPAQ scores. In contrast, BPAQ scores were not significantly associated with bone strength at tibia sites in young girls, as compared to the modified PYPAQ [6]. There are a limited number of studies that focused on the relationships between the BPAQ algorithms and bone quality parameters measured by pQCT in different age groups. Based on our findings, BPAQ scores may be more significant indices to predict aBMD and bone strength in healthy young women, but not in middle-aged premenopausal women.

There are several limitations to our study. It is possible that participants’ recall errors (e.g., lifetime recall) would affect the individual pBPAQ scores, and this error might be more evident in the older premenopausal women as compared to the young women. Our cross-sectional study design does not determine cause and effect relationships. The menstrual cycle status of our participants was determined by self-report and not verified by serum hormone measurements; thus, some participants could have been perimenopause. We did not collect blood and tissue samples, which limits the ability to explore underlying mechanisms.

The total BPAQ score-derived physical activity was a stronger predictor of bone strength and geometry as well as aBMD of femoral sites in young women as compared to middle-aged premenopausal women. Differences in age and site-specific aBMD may also contribute to the interpretation of the BPAQ algorithm. Future intervention studies are needed to further clarify these differences and their implications comparing and contrasting results obtained from objective physical activity assessment tools.
